# Fibronectin 1B Gene Plays an Important Role in Loach Barbel Air-Breathing

**DOI:** 10.3390/ijms222111928

**Published:** 2021-11-03

**Authors:** Bing Sun, Songqian Huang, Longfei Huang, Lijuan Yang, Jian Gao, Xiaojuan Cao

**Affiliations:** 1 Engineering Research Center of Green development for Conventional Aquatic Biological Industry in the Yangtze River Economic Belt, Ministry of Education, College of Fisheries, Huazhong Agricultural University, Wuhan 430070, China; sunbing931014@163.com (B.S.); huangsongqian0115@gmail.com (S.H.); atyanglijuan@163.com (L.Y.); 2 Department of Aquatic Bioscience, Graduate School of Agricultural and Life Sciences, The University of Tokyo, Bunkyo, Tokyo 113-8657, Japan; 3 Key Lab of Freshwater Animal Breeding, Ministry of Agriculture, College of Fisheries, Huazhong Agricultural University, Wuhan 430070, China; 15207173315@163.com

**Keywords:** *Misgurnus anguillicaudatus*, barbel, air-breathing, transcriptome, *fn1b*

## Abstract

Loach (*Misgurnus anguillicaudatus*) is well known to perform air-breathing through the posterior intestine and skin. However, we find here for the first time a unique central vascular structure in the loach barbel, with a blood–gas diffusion distance as short as that of the posterior intestine. Under acute hypoxia, the distance of loach barbels became significantly shorter. Moreover, barbel removal significantly decreased air-breathing frequency of the loach. These findings imply that the barbel is another air-breathing organ of the loach. For further investigation of loach barbel air-breathing, a transcriptome analysis of barbels with air exposure treatment was performed. A total of 2546 differentially expressed genes (DEGs) between the T-XU (air exposure) and C-XU (control) group were identified, and 13 key DEGs related to barbel air-breathing were screened out. On this foundation, sequence, expression, and location analysis results indicated an important positive role of fibronectin 1b (*fn1b*) in loach barbel air-breathing. We further generated an *fn1b*-depletion loach (MT for short) using the CRISPR/Cas9 technique. It was indicated that depletion of *fn1b* could weaker barbel air-breathing ability. In conclusion, due to nonlethal and regenerative characteristics, the loach barbel, a newly discovered and *fn1b*-related fish air-breathing organ, can be a good model for fish air-breathing research.

## 1. Introduction

Since fish air-breathing is an important evolutionary adaptation for aquatic hypoxia, it has fascinated researchers for at least a century [1,2,3]. Up to now, about 400 species of fish have been reported to have the ability of assisted air-breathing [4]. There are several different fish air-breathing organs reported, including modified swim bladder, oropharyngeal cavity, skin, stomach, and intestine [3,4], which share similar features, such as being well-vascularized [5] and a short blood–gas diffusion distance [6], similar to the features of higher vertebrate lungs. It has been reported that the blood–gas diffusion distance of the intestine in *Hoplosternum thoracatum* is 1–2 μm [7], of the stomach in *Ancistrus multispinis* is 0.6 μm [8], and of the intestine in *Paramisgurnus dabryanus* is 1.95 μm [9]. Table S1 summarizes the distance of air-breathing organs in six air-breathing fish [7,8,9,10,11,12]. In general, the blood–gas diffusion distance of the assistant air-breathing organs in fish is less than 3 μm [13,14].

So far, studies on air-breathing fish have mainly focused on histology, respiratory physiology, aquaculture, and evolutionary biology [15,16,17,18]. In recent years, along with the rapid development of sequencing, a very small number of studies have been performed on the transcriptome, microRNAome, and genome to reveal the molecular mechanisms of fish air-breathing formation and regulation [19]. However, all these studies remain at the stage of massive data filtering. In our previous study, a transcriptome analysis of the posterior intestine (an air-breathing organ) in loach (*Misgurnus anguillicaudatus*) with air-breathing inhibition was performed, focusing on the pathways of angiogenesis and vascular development [20]. Through comparative transcriptome analysis, some genes (for example, fibronectin 1 (*fn1*), integrin alpha (*itga*), and vascular endothelial growth factor (*vegf*)) potentially related to air-breathing were screened out from four air-breathing fishes, namely, snakehead fish (*Channa argus*) [21], channel catfish (*Ictalurus punctatus*), blue catfish (*I. furcatus*), and their F1 hybrid [22]. Due to the slow development of in vivo gene editing research in air-breathing fish and the lack of good models of air-breathing organs (with nonlethal characteristics) in fish, there are almost no key genes related to air-breathing reported. Certainly, fish air-breathing molecular mechanisms cannot really be unveiled.

The loach *M. anguillicaudatus*, an important economic fish species in many Asian countries including China, Japan, and South Korea [23], has attracted the attention of scholars because of its air-breathing for many years [19,24]. With the aid of assisted air-breathing, the demersal loach can survive well in hypoxic environments. The loach must conduct air-breathing even in air-saturated water [25]. Taking it as a model, our laboratory has carried out studies on fish air-breathing mechanisms for more than 10 years [19,20,26]. The existing studies indicate that the loach has more than one assisted air-breathing organ (i.e., posterior intestine and skin), which is rare. Does the loach have other air-breathing organs to adapt to low oxygen environment? In other words, is there another organ with a rich capillary network and a short gas diffusion distance, similar to the posterior intestine and skin in the loach, which can conduct air-breathing? There is a viewpoint that the fish barbel is a subsidiary extended structure of the skin [27,28]. Therefore, can the developed barbels in the loach perform air-breathing?

In this study, we firstly compared the histological structures of barbels among loach *M. anguillicaudatus* (a water–air bimodal respiration fish), large-scale loach (*P. dabryanus*, a water–air bimodal respiration fish which can conduct air-breathing through its posterior intestine), zebrafish (*Danio rerio*, an aquatic breather), and yellow catfish (*Pelte*o*bagrus fulvidraco*, an aquatic breather). Meanwhile, the barbel blood–gas diffusion distance and air-breathing frequency estimations, barbel excision, and acute hypoxia experiments were performed to examine the loach barbel air-breathing ability. Then, an air exposure experiment was carried out for barbel transcriptome analysis. Accordingly, sequence, expression, and location analyses were conducted to finally screen out the key air-breathing related gene, namely, *fn1b*. At last, an *fn1b*-mutant loach was generated by CRISPR/Cas9 for the first time to further confirm the role of *fn1b* in loach barbel air-breathing. This study suggests that the loach barbel is a newly discovered fish air-breathing organ, and *fn1b* is closely involved in its air-breathing function. Due to the nonlethal and regenerative characteristics of fish barbels [29,30] and the high efficiency of in vivo gene editing in loach *M. anguillicaudatus* [31], the loach barbel will be a good model for mechanistic investigations of fish air-breathing.

## 2. Results

### 2.1. Histological Structures of Loach Barbels Suitable for Air-Breathing

**Figure 1:**
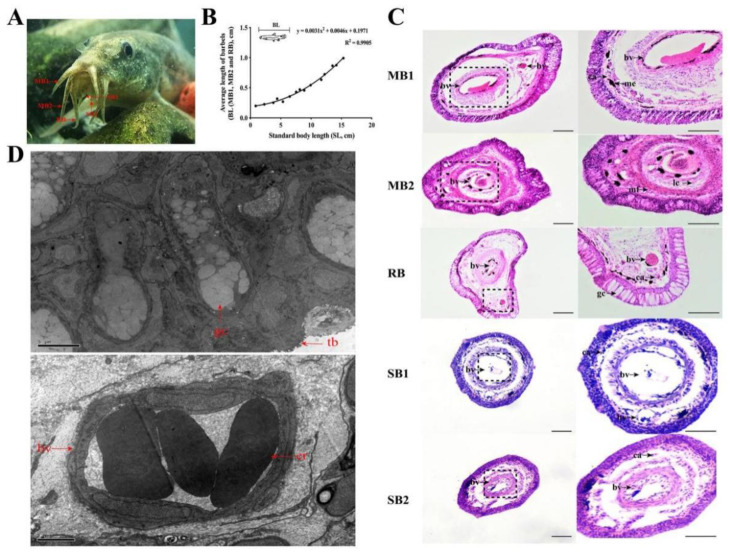
Morphological and histological observations of loach barbels. (**A**) Visual picture of loach barbels. (**B**) Linear regression curve of the average length of barbels including MB1, MB2, and RB (ordinate) and standard body length (SL, abscissa) in loach. (**C**) Observations of H&E-stained transverse sections of loach barbels. The visual fields in the black dotted boxes are magnified to the right. The scale bars in the left and right columns of transverse sections of loach barbels are 30 μm and 100 μm, respectively. (**D**) Transmission electron microscopy observation results of loach barbels. The scale bars of the top and bottom pictures are 5 μm and 2 μm, respectively. MB, maxillary barbel; RB, rostal barbel; SB, submaxillary barbel; H&E, hematoxylin and eosin; bv, blood vessels; er, erythrocytes; ca, capillaries; mf, muscle fiber; me, melanocytes; lc, loose connective tissue; gc, goblet cells; tb, taste buds.

Two pairs of maxillary barbels (recoded as MB1 and MB2), one pair of rostal barbels (RB), and two pairs of submaxillary barbels (SB1 and SB2) were observed in loach *M. anguillicaudatus* (Figure 1A). In the loach, SB1 and SB2 were very short, while the other three barbels (i.e., MB1, MB2, and RB) were large. A linear regression analysis result demonstrated that there was a highly positive correlation between the average length of barbels (including MB1, MB2, and RB) and body length of the loach (Figure 1B). Figure 1C and Figure S1 show that the five pairs of barbels in the loach (MB1, MB2, RB, SB1, and SB2) had similar histological structures. In addition to goblet cells, melanocytes, and taste buds, a large number of erythrocytes enriched in the central blood vessel and many capillaries, the structural basis of air-breathing, were found in the loach barbels. This was consistent with the transmission electron microscopy observation results (Figure 1D).

**Figure 2:**
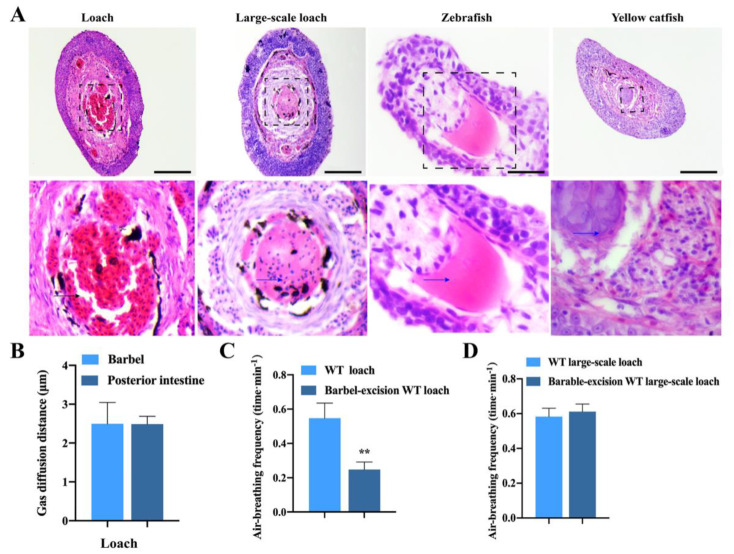
Comparisons of barbel histological structures, gas diffusion distances, and air-breathing frequency. (**A**) Comparison of histological structures of barbels among loach, large-scale loach, zebrafish, and yellow catfish. The visual fields in black dotted boxes are magnified below. The black arrow indicates erythrocytes, and the blue arrows indicate cartilage tissues. Scale bar = 100 μm. (**B**) Comparison of gas diffusion distance between the barbel and posterior intestine of loach. (**C**) Comparison of air-breathing frequency between WT and barbel-excised loach. (**D**) Comparison of air-breathing frequency between WT and barbel-excised large-scale loach. WT, wild-type. ** very significant difference (*p* < 0.01).

There was an obvious difference in barbel histological structures between the loach and other three fishes (one water–air bimodal respiration fish and two aquatic breathers). Unlike the large blood vessel found in the loach barbel center, cartilage tissues existed in the centers of barbels from the other three fishes including the large-scale loach, zebrafish, and yellow catfish (Figure 2A). Figure 2B shows no significant difference in blood–gas diffusion distance between the barbel (2.495 ± 0.194 μm) and posterior intestine (2.488 ± 0.0997 μm) of the loach, suggesting that loach barbels are suitable for air-breathing to some extent. After removing the barbels, the movement of the loach became slow. The air-breathing frequency of the loach with barbel excision was significantly lower than that of the wild-type (WT) loach (Figure 2C). However, there was no significant difference in air-breathing frequency between the WT and barbel-excised large-scale loach (Figure 2D), and barbel excision had no effect on its movement.

### 2.2. The Changes in Histological Structures and Gas Diffusion Distances of Loach Barbels in Response to Acute Hypoxia

**Figure 3:**
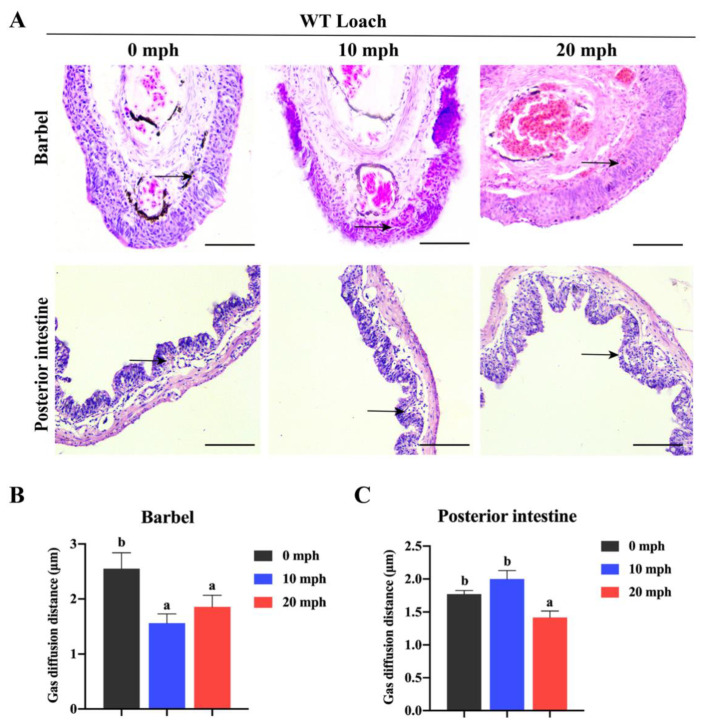
Histological structures and gas diffusion distance of barbels and posterior intestines of loach with acute hypoxia treatment. (**A**) Histological structures of barbels and posterior intestines of loach with acute hypoxia treatment. mph, minute post hypoxia. Black arrows indicate capillaries. Scale bar = 100 μm. (**B**,**C**) The gas diffusion distances of barbels (**B**) and posterior intestines (**C**) of loach with acute hypoxia treatment. Different letters above error bars indicated significant a difference among different groups (*p* < 0.05).

Along with the increase in acute hypoxia treatment time, the capillaries of loach barbels and posterior intestines extended to the dermal layer and mucosal layer, respectively (Figure 3A). For loach barbels, the blood–gas diffusion distance at 10 and 20 min post hypoxia (mph) was significantly lower than that without hypoxia treatment (i.e., at 0 mph) (Figure 3B). Similarly, the gas diffusion distance in the posterior intestine at 20 mph was significantly lower than that at 0 and 10 mph (Figure 3C).

### 2.3. Transcriptome Analysis of Loach Barbels with Air Exposure

After assembly of the mapped reads of six cDNA libraries, a total of 250.54 million raw reads and 247.26 million clean reads were obtained. The percentage of mapped clean reads ranged from 82.82% to 85.82%. The Q30 values of more than 93.32% indicated a high quality of the sequencing data (Table S2).

#### 2.3.1. Gene Ontology (GO) and Kyoto Encyclopedia of Genes and Genomes (KEGG) Enrichment Analysis of Differentially Expressed Genes (DEGs)

**Figure 4:**
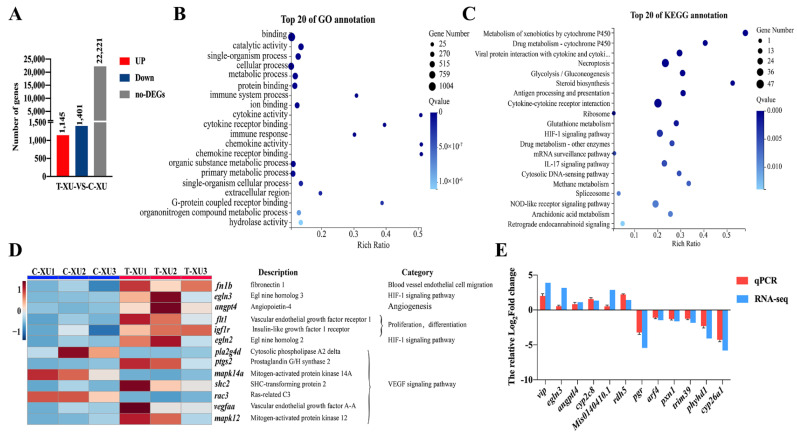
Identification and functional annotations of DEGs in barbels between T-XU and C-XU group (T-XU vs. C-XU). (**A**) Numbers of upregulated, downregulated, and non-DEGs. (**B**) Top 20 GO terms in DEGs. (**C**) Top 20 KEGG pathways in DEGs. (**D**) Heat map of expression levels of 13 key DEGs related to air-breathing. (**E**) qPCR validation of RNA-seq data. DEGs, differentially expressed genes. T-XU and C-XU represent the air exposure group and control group, respectively. qPCR, quantitative PCR. *vip*, VIP intestinal peptide; *egln3*, egl nine homolog 3; *angptl4*, angiopoietin-related protein 4; *cyp2c8*, cytochrome P450 2C8; *rdh5*, 11-*cis*-retinol dehydrogenase; *pgr*, progesterone receptor; *arf4*, ADP-ribosylation factor 4; *pxn1*, pentraxin fusion protein precursor; *trim39*, E3 ubiquitin-protein ligase TRIM39; *phyhd1*, phytanoyl-CoA dioxygenase domain-containing protein 1; *cyp26a1*, cytochrome P450 26A1.

A total of 2546 DEGs were identified, with 1145 upregulated and 1401 downregulated in the air exposure group (T-XU group) in comparison to the control group (C-XU group, without air exposure) (Figure 4A, Figure S2A). The GO analysis showed that all DEGs were classified into three categories: biological process, cellular component, and molecular function, involving 24, 15, and 11 GO terms, respectively (Figure S2B). The top 20 significantly enriched GO terms are summarized in Figure 4B. Furthermore, KEGG analysis showed that the 2546 DEGs belonged to 33 pathways, which included signal transduction (324 DEGs; 12.73%), immune system (183 DEGs; 7.19%), endocrine system (166 DEGs; 6.32%), and transport and catabolism (132 DEGs; 5.18%) (Figure S2C). Among these pathways, the top 20 significantly enriched KEGG pathways were identified (Figure 4C).

#### 2.3.2. Mining of DEGs Related to Barbel Air-Breathing

Referring to the structural needs of air-breathing function in fish (namely, rich capillary networks) and the existing research results [19,21,22], some KEGG pathways of DEGs including the HIF-1 signaling pathway, VEGF signaling pathway, and blood vessel endothelial cell migration pathway were targeted, and 13 key DEGs (*fn1b*, egl nine homolog 3 (*egln3*), angiopoietin 4 (*angpt4*), vascular endothelial growth factor receptor 1(*flt1*), insulin-like growth factor 1 receptor (*igf1r*), egl nine homolog 2 (*egln2*), cytosolic phospholipase A2 delta (*pla2g4d*), prostaglandin G/H synthase 2 (*ptgs2*), mitogen-activated protein kinase 14 a (*mapk14a*), shc-transforming protein 2 (*shc2*), ras-related C3 (*rac3*), *vegfaa*, and mitogen-activated protein kinase 12 (*mapk12*); 10 up- and three downregulated genes) were screened out (Figure 4D).

#### 2.3.3. qPCR Validation of RNA-Seq Data

To validate the RNA-seq data, we randomly selected 12 DEGs to conduct quantitative PCR (qPCR) analysis. The qPCR results were consistent with the RNA-seq data (Figure 4E).

### 2.4. Sequence and Expression Profiles of Fn1b in Loach

To further confirm the role of *fn1b* (the most prominent key DEG screened out above) in loach barbel air-breathing, a sequence analysis of FN1 was firstly performed. The FN1 conserved domains of loach, zebrafish, snakehead fish, and rice-field eel were predicted, showing that the FN1a protein was conserved in fish, while the FN1b protein of loach (with one FN3 module less) was very different from the other three fishes (Figure S3). This suggested that the *fn1b* gene is special in loach.

**Figure 5:**
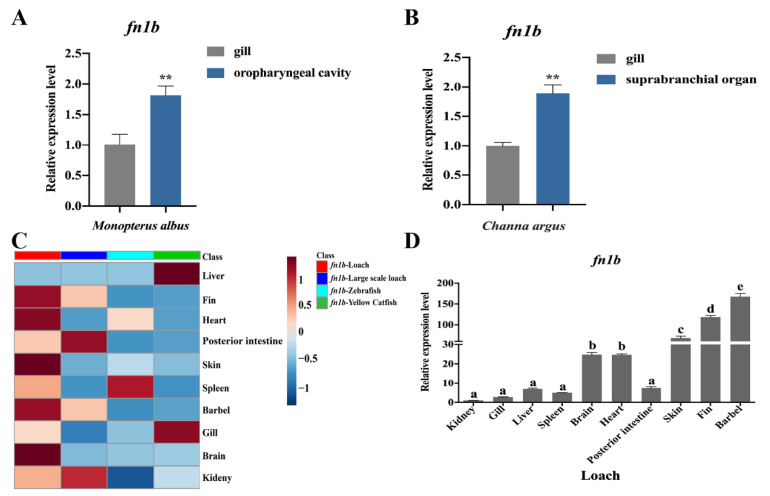
*fn1b* expression levels. (**A**) Expression levels of *fn1b* in the gill (aquatic breathing organ) and oropharyngeal cavity (an air-breathing organ) of rice-field eel *Monopterus albus* were calculated using the 2^−ΔΔCT^ method. *β-actin* was used as the reference gene for normalization. (**B**) Expression levels of *fn1b* in gill and suprabranchial organ (an air-breathing organ) from snakehead fish *Channa argus* were calculated using the 2^−ΔΔCT^ method. *β-actin* was used as the reference gene for normalization. (**C**) Tissue expression levels of *fn1b* in loach, large-scale loach, zebrafish, and yellow catfish. MetaboAnalyst5.0 was used for constructing the heat map. (**D**) Comparison of *fn1b* relative expression across different tissues from the loach (*Misgurnus anguillicaudatus*). Tissue expression levels of *fn1b* in loach were calculated using the 2^−ΔΔCT^ method. *β-actin* and *gapdh* were used as reference genes for normalization. *fn1b*, fibronectin 1b; *gapdh*, glyceraldehyde-3-phosphate dehydrogenase. Different letters above error bars indicate significant difference among different tissues (*p* < 0.05). ** very significant difference (*p* < 0.01).

Next, Figure 5A,B show that the expression level of *fn1b* in the air-breathing organ (oropharyngeal cavity in rice-field eel and suprabranchial organ in snakehead fish) was significantly higher than that of the aquatic breathing organ (gill). In addition, *fn1b* was also found highly expressed in the posterior intestine (an air-breathing organ) of large-scale loach (Figure 5C). These results suggest that *fn1b* may be highly expressed in many air-breathing organs in fish. Compared with large-scale loach (air-breathing fish), zebrafish (aquatic breathing fish), and yellow catfish (aquatic breathing fish), the loach presented an obviously higher expression of *fn1b* in the barbels (Figure 5C). In addition, compared with the kidney, about 68-, 118-, and 167-fold higher *fn1b* expression was detected in the skin, fin, and barbel (air-breathing organs) of loach, respectively (Figure 5D). This indicated that *fn1b* plays a positive role in loach barbel air-breathing.

**Figure 6:**
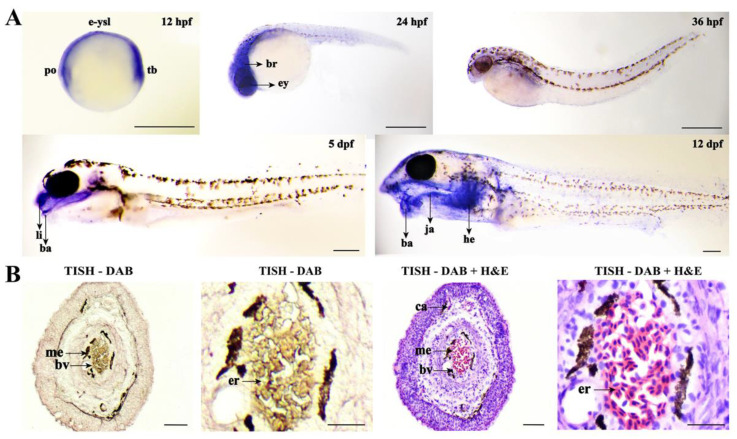
The localization of *fn1b* in loach. (**A**) Whole-mount in situ hybridization of *fn1b* in loach embryos of different developmental stages, including 12 hpf, 24 hpf, 36 hpf, 5 dpf, and 12 dpf. hpf, hours post fertilization; dpf, days post fertilization; po, polster; tb, tail bud; e-ysl, external yolk syncytial layer; br, brain; ey, eye; li, lip; ba, barbels; he, heart; ja, jaw. Scale bar = 1 mm. (**B**) Tissue in situ hybridization (TISH) of *fn1b* in loach barbels. Brown color signifies the signals. DAB, 3,3′-diaminobenzidine tetrahydrochloride; H&E, hematoxylin and eosin; bv, blood vessel; me, melanocytes; er, erythrocytes; ca, capillary. The scale bars of the left and the right pictures indicated 100 μm and 10 μm, respectively.

Then, whole-mount in situ hybridization (WISH) and tissue in situ hybridization (TISH) were performed to locate the *fn1b* gene in loach. Figure 6A showed that *fn1b* was highly expressed in the polster, tail bud, and external yolk syncytial layer of loach embryos at 12 h post fertilization (hpf), in the eye and brain of loach embryos at 24 hpf, and in the lip and barbel of the loach at 5 days post fertilization (dpf). At 12 dpf, the expression signals were mainly concentrated in the barbel, jaw, and heart. Furthermore, the TISH results indicated that *fn1b* was located in blood vessels of loach barbels (Figure 6B).

### 2.5. The Depletion of Fn1b Weakened the Air-Breathing of Loach Barbel

The chromatogram of MT loach (*fn1b^+/−^* loach) showed that a C base was missing at exon 3 of the *fn1b* gene, which led to premature translation termination (the original translation of 2398 amino acids was terminated at the 135th) (Figure S4A). The survival rate of F2 generation fertilized eggs (F1 generation self-crossed) was obviously lower than that of F1 generation (F0 generation crossed with WT loach) and WT loach fertilized eggs (Figure S4B), which to some extent could explain the failure of *fn1b^−/−^* generation in this study. Figure S4C shows that the expression level of *fn1b* in MT loach was significantly lower than that of WT loach.

**Figure 7:**
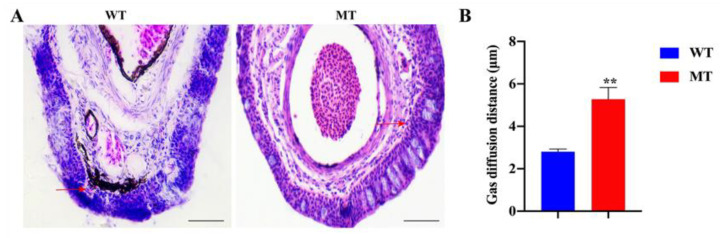
Histological structures (**A**) and gas diffusion distances (**B**) of barbels in WT and MT loach. Red arrows show capillaries. WT, wild-type loach; MT, *fn1b-*depletion loach. Scale bar = 100 μm. ** very significant difference (*p* < 0.01).

**Figure 8:**
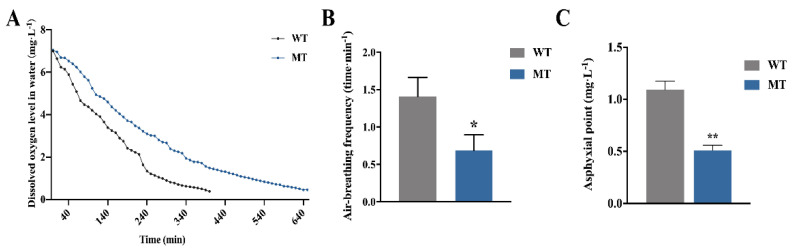
The effects of *fn1b*-depletion on air-breathing function of WT and MT loach under chronic hypoxia. (**A**) Water dissolved oxygen levels in the sealed conical flasks with WT and MT loach. (**B,C**) Comparison of air-breathing frequency (**B**) and asphyxia point (**C**) between WT and MT loach under chronic hypoxia. WT, wild-type loach; MT, *fn1b*-depletion loach. * significant difference (*p* < 0.05); ** very significant difference (*p* < 0.01).

Figure 7A shows the histological structures of barbels of WT and MT loach. It is worth noting that the barbel blood–gas diffusion distance of MT loach was significantly longer than that in WT (Figure 7B). Under chronic hypoxia, we found that the movement of MT loach was relatively slow compared with WT loach. Figure 8A indicates that the water oxygen consumption of MT loach with chronic hypoxia treatment was clearly lower than that of WT loach. Meanwhile, the air-breathing frequency and the asphyxia point of MT loach were significantly lower than those of WT loach (Figure 8B,C).

## 3. Discussion

Many fish have barbels. The number of barbels in fish varies from species to species. For example, *Osteogeniosus bleeker* [32] has only one pair of barbels. Both common carp (*Cyprinus carpio*) [33] and zebrafish [28] possess two pairs of barbels. This study shows that the number of barbels in loach is up to five pairs. The fish barbel is generally considered as a sensory organ, which fish can use to find and choose food. The barbel structures are simple, normally composing of a central rod of cartilage, connective tissue, taste buds, and nerve trunk [30]. Interestingly, in this study, we found a unique central vascular structure in loach barbel, very different from the axial cartilage tissues in other aquatic-breathing and air-breathing fishes. Loach barbels are well-vascularized, which is the structural basis of air-breathing [34]. In addition, the blood–gas diffusion distance of the barbel was as short as that of the posterior intestine (a confirmed air-breathing organ) in loach. The blood–gas diffusion distance is an important indicator of air-breathing [3]. A short blood–gas diffusion distance is a structural adaptation of air-breathing organs, which can greatly enhance the efficiency of gas exchange [34]. Under acute hypoxia, the blood–gas diffusion distance of loach barbels became significantly shorter, and this was also the case for the loach posterior intestine, as well as the air-breathing organs of *H. thoracatum* [7] and *Boleophthalmus boddaerti* [35]. After removing barbels, a significant decrease was observed in loach air-breathing frequency. Considering these characteristics of loach barbels above (namely, the unique vascular structures, short blood–gas diffusion distance, and air-breathing behavior), it is reasonable to believe that the loach barbel is a newly discovered air-breathing organ.

Currently, transcriptome analysis is widely used to explore molecular mechanisms of important life activities [36,37] In this study, a transcriptome analysis of loach barbels with air exposure treatment was performed, and some KEGG pathways and key genes related to barbel air-breathing were screened out. Among them, the angiogenesis and vascular development pathway, HIF-1 signaling pathway, and VEGF pathway were prominent. These pathways were also identified in previous studies on air-breathing of loach [19] and snakehead fish [21]. In this study, the blood vessel morphogenesis- and development-related genes (for example, *fn1b* and *vegfaa*) were remarkably induced in loach barbels with air exposure, suggesting that *fn1b* and *vegfaa* may be closely related to fish air-breathing organs.

It has been reported that *fn1* is crucial for blood vessel development and heart morphogenesis in both zebrafish [38] and mouse [39,40]. In this study, *fn1b* showed a significantly high expression in loach barbels based on qPCR and ISH analysis. Moreover, the expression levels of *fn1b* in air-breathing organs of rice-field eel and snakehead fish were significantly higher than in gills, as also found in a previous study reported by Jiang et al. [21]. Therefore, these results further indicated that *fn1b* is important for loach barbel air-breathing.

For exploring the role of *fn1b* in air-breathing of loach barbels, an *fn1b*-mutant loach line was generated for the first time using the CRISPR/Cas9 technique. Compared with WT loach, MT loach possessed a longer blood–gas diffusion distance in barbels, suggesting that *fn1b* depletion weakened the air-breathing ability of loach barbels. In addition, under chronic hypoxia condition, the oxygen consumption, air-breathing frequency, and asphyxia point of MT loach were significantly decreased compared with WT loach. As we know, oxygen consumption is positively correlated with fish movement [41,42]. The low oxygen consumption and air-breathing frequency in MT loach would lead to its weakened air-breathing ability, so as to maintain slow movement. These findings again suggested that *fn1b* plays a positive role in barbel air-breathing of loach.

Reports related to the fish *fn1* gene and its encoded fibronectin (FN) were mainly generated in zebrafish. There are two subtypes of *fn1a* and *fn1b* in zebrafish [43] and loach. Interestingly enough, unlike other fish species (including zebrafish, rice-field eel, and snakehead fish), the FN1b protein of loach lacks an FN3 module. FN3 is the most abundant and common domain of the FN1 protein, which has important functional and structural properties in angiogenesis [44]. The special structure of loach FN1b suggests that it may perform different functions, which needs further investigations.

In conclusion, the present study strongly suggests that barbels play an important role as air-breathing organs, which has never been identified, particularly in loach. Moreover, using loach barbel as a new model for air-breathing in fishes, we clearly show that *fn1b* plays a crucial role in this process. As we propose the barbel as an additional air-breathing organ in fish, there are even more important gene analysis studies to conduct (fibronectin supramolecular complex) in sensory cells (electrophysiological responses of barbel cells to air or airborne molecules). These new perspectives opened up by our work are particularly important when it comes to further characterization of the functional importance of barbels in sensing the complex environment of a fish, such as hypoxia.

## 4. Materials and Methods

### 4.1. Fish Species

All experimental diploid WT loaches *M. anguillicaudatus* (body length of 10–12 cm; body weight of 9–10 g; 1 year old) were obtained from the Aquaculture Base of College of Fisheries, Huazhong Agricultural University in Wuhan City, Hubei Province, China. Large-scale loach (11–12 cm; 13–15 g; 1 year old), yellow catfish (10–13 cm; 110–130 g; 1 year old), rice-field eel (*Monopterus albus*, a water–air bimodal respiration fish which can conduct air-breathing through its oropharyngeal cavity; 18–20 cm; 150–170 g; 1 year old), and snakehead fish (a water–air bimodal respiration fish which can conduct air-breathing through its suprabranchial organ; 19–21 cm; 240–280 g; 1 year old) were obtained from the Baishazhou aquatic products market. Zebrafish (AB strain, 0.4–0.5 g; 4–6 cm; 2 months old) were purchased from the Institute of Hydrobiology, Chinese Academy of Sciences, China Zebrafish Resource Center, Wuhan, China.

### 4.2. Morphological and Histological Observations and Estimations of Blood–Gas Diffusion Distance and Air-Breathing Frequency

Morphological observation of WT loach barbels was performed, and the correlation of barbel length and body length was estimated. Barbels sampled from WT loach, large-scale loach, zebrafish, and yellow catfish were fixed in 4% paraformaldehyde for 24 h, dehydrated in graded ethanol, and embedded in paraffin wax. Cross-sections of 5 μm thickness were stained with hematoxylin and eosin (H&E) and then prepared for light microscopy, according to the method of Cao and Wang [45]. Next, the blood–gas diffusion distance, the shortest distance between the capillary and the epidermis, was measured with the use of Image J software (National Institutes of Health, Bethesda, MD, USA). Meanwhile, H&E staining of the posterior intestine tissue section of the loach was performed for blood–gas diffusion distance assessment.

For transmission electron microscopy, the barbel tissues (MB1, size: 1.0 mm × 1.0 mm × 1 mm) were fixed immediately in fixative, post-fixed in osmium tetroxide, and embedded in Spurr’s resin. The section was performed by the platform of transmission electron microscopy of Huazhong Agricultural University. The microphotograph was taken using a transmission electron microscope (HT7700, HITACHI, Tokyo, Japan).

Three WT loaches and three barbel-excised loaches of the same size were placed into a 2 L conical flask containing 1.5 L of water (initial dissolved oxygen (DO): 6.0 mg/L) (Figure S5A, normoxia treatment) to measure the air-breathing frequencies, which were recorded within 2 h. At the same time, the swimming behaviors of the loaches were observed. Three parallel experiments were carried out. Similarly, the air-breathing frequencies of healthy WT and barbel-excised large-scale loach were measured.

To further explore the loach barbel air-breathing ability, an acute hypoxia experiment (Figure S5B) was performed. WT loaches were placed into 2 L conical flasks containing 1 L of water, whilst nitrogen was poured into the flasks to drastically decrease its dissolved oxygen content. Loach barbels and posterior intestines were sampled at 0, 10, and 20 mph for histological analysis. Then, the blood–gas diffusion distances of both kinds of tissues were calculated.

### 4.3. Transcriptome Analysis of Loach Barbel

#### 4.3.1. Air Exposure Experiment

A total of 18 WT loaches were used here. The loaches were cultured in a 25 L glass tank with 20 L of water (temperature: 24.0 to 26.0 °C; pH: 7.0 to 7.5; DO: 6.0 mg/L). Among them, nine loaches were randomly selected for barbel sampling, namely, the control group (C-XU). Then, the remaining nine loaches were placed on moist towels (air exposure group (T-XU), Figure S5C). Based on our pre-test results, an air exposure time of 6 h was applied. Barbels sampled from C-XU and T-XU group were stored at −80 °C for RNA isolation.

#### 4.3.2. RNA Isolation and cDNA Library Constructions

Total RNA was isolated from the barbel tissues using TRIzol Reagent (TaKaRa, Dalian, China) according to the manufacturer’s protocol. The extracted RNA samples of high quality were used for cDNA synthesis. Poly (A) mRNA was isolated using oligo-dT beads (Qiagen). All mRNA was sheared into short fragments (200 nt) by adding fragmentation buffer. First-strand cDNA was generated using random hexamer-primed reverse transcription, followed by the synthesis of the second-strand cDNA using RNase H and DNA polymerase I. The cDNA fragments were purified using a QIAquick PCR extraction kit. These purified fragments were then washed with EB buffer for end reparation poly (A) addition and ligated to sequencing adapters. Following agarose gel electrophoresis and extraction of cDNA from gels, the cDNA fragments (200 ± 5 bp) were purified and enriched by PCR to construct the cDNA library (PE100). Six cDNA libraries were constructed (namely, C-XU-1, C-XU-2, C-XU-3, T-XU-1, T-XU-2, and T-XU-3) in this study.

#### 4.3.3. Sequencing and Read Mapping

The cDNA libraries were sequenced on an Illumina platform (Illumina Hiseq xten/NovaSeq6,000 sequencer Illumina, San Diego, CA, United States) and 150 bp paired-end reads were generated. The raw paired-end reads were trimmed and quality-controlled using SeqPrep (https://github.com/jstjohn/SeqPrep, accessed on 11 August 2021) and Sickle (https://github.com/najoshi/sickle, accessed on 11 August 2021) with default parameters. Then, clean reads were separately aligned to the loach genome (unpublished) using HISAT2 (http://www.ccb.jhu.edu/software/hisat, accessed on 11 August 2021) [46] software. The mapped reads of each sample were assembled by StringTie (https://ccb.jhu.edu/software/stringtie/index.shtml? t = example, accessed on 11 August 2021) in a reference-based approach [47].

#### 4.3.4. Identification of DEGs and Functional Annotations

To identify DEGs between the two different groups (T-XU and C-XU), the expression level of each transcript was calculated according to the transcripts per million reads (TPM) method. RSEM (http://deweylab.biostat.wisc.edu/rsem/, accessed on 11 August 2021) [48] was used to quantify gene abundances. Differential expression analysis was performed using DESeq2. Genes with |log_2_ fold change| ≥ 1 and false discovery rate (FDR) < 0.05 were considered to be significantly differentially expressed genes [49]. In addition, GO functional enrichment analysis and KEGG pathway analysis were carried out using Goatools (https://github.com/tanghaibao/Goatools, accessed on 11 August 2021) and KOBAS (http://kobas.cbi.pku.edu.cn/home.do, accessed on 11 August 2021) [50].

#### 4.3.5. qPCR Validation of Transcriptome Data

To examine the reliability of the transcriptome results, we randomly selected 12 DEGs (six upregulated genes: VIP intestinal peptide (*vip*), *egln3*, angiopoietin-related protein 4 (*angptl4*), cytochrome P450 2C8 (*cyp2c8*), *mis0140410.1*, and 11-*cis*-retinol dehydrogenase (*rdh5*); six downregulated genes: progesterone receptor (*pgr*), ADP-ribosylation factor 4 (*arf4*), pentraxin fusion protein precursor (*pxn1*), E3 ubiquitin-protein ligase TRIM39 (*trim39*), phytanoyl-CoA dioxygenase domain-containing protein 1 (*phyhd1*), and cytochrome P450 26A1 (*cyp26a1*)) to conduct qPCR analysis. Total RNAs of the barbel samples from C-XU and T-XU group were extracted using RNA isoPlus (TaKaRa, Dalian, China). qPCR was carried out on a QuantStudioTM 3 Real-time PCR system (ThermoFisher) according to the manufacturer’s instructions. qPCR conditions were as follows: 95 °C for 30 s followed by 40 cycles consisting of 95 °C for 5 s and 57 °C for 30 s. The fluorescent flux was then recorded, and the reaction continued at 72 °C for 6 s and 95 °C for 5 s. Finally, the relative expression levels were normalized to the endogenous control gene *β-actin* and glyceraldehyde-3-phosphate dehydrogenase (*gapdh*) and calculated using the 2^−ΔΔCT^ method. All procedures were based on the methods described by Liu et al. [51]. The relative expression levels of these genes (log_2_ fold change (T-XU vs. C-XU)) observed by qPCR and RNA-seq analyses were used for graphical presentation. All of the primer sequences for qPCR are listed in Table S3.

### 4.4. Sequence, Expression, and Location Analyses of fn1b

#### 4.4.1. Sequence Analysis

To further confirm the role of *fn1b* gene (a key DEG screened out from the transcriptome analysis above) in loach barbel air-breathing, a sequence analysis was firstly conducted. Deduced amino-acid sequences of *fn1* genes in three other fish species (zebrafish, rice-field eel, and snakehead fish) were obtained from the NCBI database. The conserved domains were analyzed using Conserved Domains (https://www.ncbi.nlm.nih.gov/Structure/cdd/wrpsb.cgi, accessed on 20 August 2021), and a schematic diagram was generated by Adobe Illustrator CC 2019 software (Adobe, San Jose, CA, USA).

#### 4.4.2. qPCR Analysis

Ten tissues (i.e., kidney, gill, liver, spleen, brain, heart, posterior intestine, skin, fin, and barbel) were sampled from WT loach, large-scale loach, zebrafish, and yellow catfish. All samples were used for qPCR analysis, and the expression levels of *fn1b* in different tissues from the four kinds of fishes were analyzed by MetaboAnalyst5.0 (https://www.metaboanalyst.ca, accessed on 20 August 2021). In addition, gills (aquatic breathing organ) were sampled from rice-field eel and snakehead fish. The air-breathing organs, oropharyngeal cavity and suprabranchial organ, were dissected from rice-field eel and snakehead fish, respectively. All samples were used for qPCR analysis, and *β-actin* was used as the reference gene. All of the primer sequences for qPCR are listed in Table S3.

#### 4.4.3. In Situ Hybridization

To locate the *fn1b* gene in the loach barbel, a specific Dig-labeling antisense RNA probe was synthesized by using T7 in vitro transcriptional polymerase with a DIG RNA labeling kit (Roche Molecular Biochemicals, Mannheim, Germany). The probe for *fn1b* location was amplified from the cDNA pool using appropriate primers (Table S3). A healthy WT loach was selected for reproduction. Development of embryos occurred at 26 °C, and staging was determined by hours post fertilization. Living loach embryos were sampled at different developmental stages: 12 h post fertilization (hpf), 24 hpf, 36 hpf, 5 days post fertilization (dpf), and 12 dpf. All embryos were fixed in 4% (*w*/*v*) paraformaldehyde. Then, whole-mount in situ hybridization (WISH) of loach embryos was conducted according to a previously described method [30]. As for tissue in situ hybridization (TISH), barbels from WT loach were sampled and fixed in 4% (*w*/*v*) paraformaldehyde. The procedure of TISH was performed as described previously [52].

### 4.5. Effects of fn1b-Depletion on Loach Barbel Air-Breathing

#### 4.5.1. fn1b^+/−^ Loach Produced by CRISPR/Cas9

The *fn1b*-depletion loach was generated using CRISPR/Cas9 technology. The Cas9 targeting site design website was used to design the appropriate targeting site (http://zifit.partners.org/ZiFi/, accessed on 20 August 2021). Following all design principles, the Cas9 targeting site “GATTGTACCTGCATTGGGTCAGG” was determined. In vitro transcription of Cas9 RNA and gRNA was based on the standards of relevant research [53], and detailed construction methods were performed as previously described [54]. After microinjection, the genomic DNA was isolated from six randomly selected fertilized embryos. Next, the target genome region was amplified and sequenced. The primers for mutation analysis are listed in Table S3. Once the mutation was confirmed in injected embryos, the remaining ones were raised to adulthood, and the mutant ones (F0 generation) were outcrossed with WT loach to produce the F1 generation. The heterozygous F1 generation with the same mutation sequences was self-crossed. During the period, the survival rates of loach fertilized eggs were recorded. Since homozygous mutants (lethal) could not be obtained, the *fn1b^+/−^* loach (*fn1b*-depletion loach, MT) was used for further analysis. The expression levels of *fn1b* in barbels of WT and MT loach were measured.

#### 4.5.2. Histological Observations of Barbels from WT and MT Loach

The barbels were collected from WT and MT loach. H&E staining of a barbel tissue section was performed, and barbel blood–gas diffusion distances were measured.

#### 4.5.3. Chronic Hypoxia Experiment

A chronic hypoxic experiment was performed to explore the effects of *fn1b*-depletion on loach air-breathing (Figure S5D). Three WT and three MT loaches were placed into a 2 L conical flask containing 1.5 L of water. We sealed the conical flask to continuously reduce the dissolved oxygen level, and the water dissolved oxygen level was monitored by a dissolved oxygen detector in real time until two loaches fainted. During this process, water dissolved oxygen levels, air-breathing frequencies, and asphyxia points were recorded. At the same time, the swimming behaviors of the loaches were observed. Three parallel experiments were carried out.

### 4.6. Statistical Analysis

All data were presented as the mean ± standard deviation (SD). Statistical analyses were performed using SPSS 26.0 software (IBM Analytics, Richmond, VA, USA). For two-group comparison, a *t*-test was performed. One-way ANOVA was performed followed by Tukey’s test for multiple comparisons. A *p*-value < 0.05 was considered significant, whereas *p* < 0.01 indicated a very significant difference.

## Data Availability

Data from this study can be provided upon request.

## Supplementary Materials

The following are available online at https://www.mdpi.com/article/10.3390/ijms222111928/s1: Figure S1. Observations of H&E-staining longitudinal sections of loach barbels; Figure S2. Functional annotations of DEGs in barbels between T-XU and C-XU groups (T-XU vs. C-XU); Figure S3. Schematic diagram of the conserved domains of the deduced amino-acid sequences of fibronectin 1 gene (*fn1*) in loach *M. anguillicaudatus*, compared with three other fish species (*D. rerio*, *C. argus*, and *M. albus*); Figure S4. *fn1b*-depletion loach produced by CRISPR/Cas9 technology; Figure S5. Four treatments used in this study. Table S1. Blood–gas diffusion distances of air-breathing organs in different air-breathing fishes; Table S2. Quality and mapped ratio of clean reads; Table S3. Primers used in this study.

## Data Accessibility

Raw sequencing data were deposited in the NCBI Sequence Read Archive (SRA) under accession number PRJNA743945.
