# Supplementary material for: Fibronectin 1B Gene Plays an Important Role in Loach Barbel Air-Breathing
Source: Int J Mol Sci. 2021 Nov 3;22(21):11928. doi: 10.3390/ijms222111928 (PMC8584523; doi:10.3390/ijms222111928)
Supplement: Supplementary file 1 [file ijms-22-11928-s001.zip › ijms-1391002-proofed-suppl.pdf]

## Supplementary Materials:

Fibronectin 1b gene plays an important role in loach barbel air-breathing

Bing Sun[1#], Songqian Huang[1,3#], Longfei Huang[2], Lijuan Yang[1,2], Jian Gao[1.2*], Xiaojuan Cao[1,2*]

[1]College of Fisheries, Engineering Research Center of Green development for Conventional Aquatic Biological Industry in the Yangtze River Economic Belt, Ministry of Education, Huazhong Agricultural University, Wuhan 430070, China.
[2]College of Fisheries, Key Lab of Freshwater Animal Breeding, Ministry of Agriculture, Huazhong Agricultural University, Wuhan 430070, China.
[3]Department of Aquatic Bioscience, Graduate School of Agricultural and Life Sciences, the University of Tokyo, Bunkyo, Tokyo 113-8657, Japan.

[#] means co-first author.

*Co-corresponding author: Jian Gao

TEL: 86(027)87282113. Email address: gaojian@mail.hzau.edu.cn (J. Gao)

Postal address: No.1 Shizishan Stress, Hongshan District, Wuhan 430070, Hubei Province, China

*Co-corresponding author: Xiaojuan Cao

TEL: 86(027)87282113. Email address: caoxiaojuan@mail.hzau.edu.cn (X.J. Cao)

Postal address: No.1 Shizishan Stress, Hongshan District, Wuhan 430070, Hubei Province, China

**Table S1 Blood-gas diffusion distances of air-breathing organs in different air-breathing fishes.**

| Air-breathing fish | Air breathing organs | Diffusion distances | Resources |
|---|---|---|---|
| *Hoplosternum thoracatum* | Intestine | 1-2 μm | Huebner & Chee 1978 [7] |
| *Ancistrus multispinis* | Stomach | 0.6 μm | Satora 1998 [8] |
| *Paramisgurnus dabryanus* | Intestine | 1.95 ± 0.34 μm | Liu & Wang 2017 [9] |
| *Anabas testudineus* | Suprabranchial apparatus | 0.21 μm | Hughes & Singh 1970 [10] |
| *Channa punctate* | Suprabranchial apparatus | 0.78 μm | Hakim et al. 1978 [11] |
| *Lepidocephalichthys guntea* | Intestine | 0.86-1.08 μm | Yadav & Singh 1980 [12] |

## Table S2 Quality and mapped ratio of clean reads.

| Sample | Total raw reads (M) | Total clean reads (M) | Clean reads Q30 (%) | Total mapping (%) |
|---|---|---|---|---|
| C-XU-1 | 41.93 | 41.51 | 93.7 | 83.21 |
| C-XU-2 | 41.42 | 40.76 | 93.74 | 82.82 |
| C-XU-3 | 41.63 | 41.03 | 93.8 | 84.23 |
| T-XU-1 | 42.77 | 42.21 | 93.32 | 85.46 |
| T-XU-2 | 40.84 | 40.31 | 93.36 | 85.82 |
| T-XU-3 | 41.95 | 41.44 | 93.66 | 85.61 |
| Total | 250.54 | 247.26 | | |

C-XU and T-XU represented control group and air exposure group, respectively. M meant million.

**Table S3 Primers used in this study.**

| Gene name | Primer sequence (5′- 3′) |
|---|---|
| Primers of loach for qPCR | |
| *gapdh*-F | ACCAACTGCTTGGCTCCCC |
| *gapdh*-R | GGAATGACTTTGCCCACG |
| *β-actin*-F | TTCCTGGGTATGGAGTCTTGCG |
| *β-actin*-R | AGAGGTTTAGGTTGGTCGTTTG |
| *fn1b*-F | GCACCACCTCTGATTACG |
| *fn1b*-R | ACCACACACTTCATACTATC |
| *vegfa*-F | TCCTGCTGAATCTCCACCAACA |
| *vegfa*-R | TCCGCTTGTGACGCTTCTGTAT |
| *epor*-F | AACTGTGGAGGCGGAAGAG |
| *epor*-R | GTGTTGGCGAAGAGTGTCTG |
| *vip*-F | AAGCGATGCTCGTGAGGAA |
| *vip*-R | GCGGCTGTAGTTATCTGTGAAT |
| *egln3*-F | GACAGTTAGTGAGCCAGAA |
| *egln3*-R | CGCATACCTCGTAGCATAT |
| *angptl4*-F | GCTATGGCAGGCGTATCAGA |
| *angptl4*-R | TGGCACAGTTGAGGTCATTCT |
| *cyp2c8*-F | CCACAGCACGCCTTACAGA |
| *cyp2c8*-R | TCGGTTCCAGCCAAGAAGAG |
| *mis0140410.1*-F | CATTGGTCACGATGCTCTT |
| *mis0140410.1*-R | AACGCCAGACATCACAAC |
| *rdh5*-F | ATGACGCAGTTCAAGCAGTG |
| *rdh5*-R | AACAGACGCCAAGGTAGATGA |
| *pgr*-F | CAGCATTCACATACAGCCTACA |
| *pgr*-R | TTACGACCTCCAAGCATCATTC |
| *arf4*-F | CACAATCCTCTACCAACTCA |
| *arf4*-R | AACATCTCGCAACTCATCA |
| *pxn1*-F | TGCCGCCTCTTAACCTTCT |
| *pxn1*-R | TCCTGTATCTCTACCACAATGC |
| *trim39*-F | CTGCTGATGCTCTTCTGCTT |

| | |
|---|---|
| *trim39*-R | GAATCCATTCGCTGGTGTCA |
| *phyhd1*-F | CAGGCATTGGTGGAGAAGTGA |
| *phyhd1*-R | GCGAGCGATCCGATGTGTT |
| *cyp26a1*-F | GGCTCTGATACACTGTCCAATG |
| *cyp26a1*-R | TCCTCCTCGTCCGTCTTGA |
| Primers for ISH | |
| *fn1b-p*F | TGCGAGGAACACGATGGACA |
| *fn1b-p*R | (TAATACGACTCACTATAGGG)[#] GTCAAAGCGAGTGACCTCTC |
| Primers for gene mutation analysis | |
| *fn1b-g*F | CAGCCGAGGAGACGTGCTAT |
| *fn1b-g*R | GTTACCTCCTTCATGGCAGC |
| Primers of rice-field eel for qPCR | |
| *β-actin*-F | CTTGGTATGGAGTCCTGCGG |
| *β-actin*-R | ATCTTCATGGTGGATGGGGC |
| *fn1b*-F | TTAATGGACGCAACCGTGGA |
| *fn1b*-R | ACGCAGGAGGTTTGATCAGG |
| Primers of snakehead fish for qPCR | |
| *β-actin*-F | CATCGAGCACGGTATCGTCA |
| *β-actin*-R | TGTTGGCTTTGGGGTTGAGT |
| *fn1b*-F | GACTCCCACCCCATTCAGTG |
| *fn1b*-R | CAAAGCGTGTAATCTCGCGG |
| Primers of large-scale loach for qPCR | |
| *β-actin*-F | TTCCTGGGTATGGAGTCTTGCG |
| *β-actin*-R | AGAGGTTTAGGTTGGTCGTTTG |
| *fn1b*-F | GCCGTGTCCGTATCAGTGA |
| *fn1b*-R | CTGGAGGTTGGTTGGAGACT |
| Primers of zebrafish for qPCR | |
| *β-actin*-F | TTACCCACACCGTGCCCATCTAC |
| *β-actin*-R | TACCGCAAGACTCCATACCCA |
| *fn1b*-F | GGAGTGGAGAGTGAACCT |
| *fn1b*-R | GTGACGATGATAGCAGTGT |
| Primers of yellow catfish for qPCR | |
| *β-actin*-F | GCCGTGACCTGACTGACTACCT |
| *β-actin*-R | AGAGGAGGAAGAGGCAGCAGTG |

| | |
|---|---|
| *fn1b*-F | CCTCGTCGTGTTCGGATCTCCA |
| *fn1b*-R | TGGTGGCAGCAATGGTGAATGG |

qPCR, quantitative PCR; *gapdh*, glyceraldehyde-3-phosphate dehydrogenase; *fn1b*, fibronectin 1b; *vegfa*, vascular endothelial growth factor a; *epor*, erythropoietin receptor; *vip*, VIP peptides intestinal peptide; *egln3*, Egl nine homolog 3; *angptl4*, angiopoietin-related protein 4; *cyp2c8*, cytochrome P450 2C8; *rdh5*, 11-cis retinol dehydrogenase; *pgr*, progesterone receptor; *arf4*, ADP-ribosylation factor 4; *pxn1*, pentraxin fusion protein Precursor; *trim39*, E3 ubiquitin-protein ligase TRIM39; *phyhd1*, phytanoyl-CoA dioxygenase domain-containing protein 1; *cyp26a1*, cytochrome P450 26A1. ISH, *in situ* hybridization. [#] indicated T7 promoter sequences.

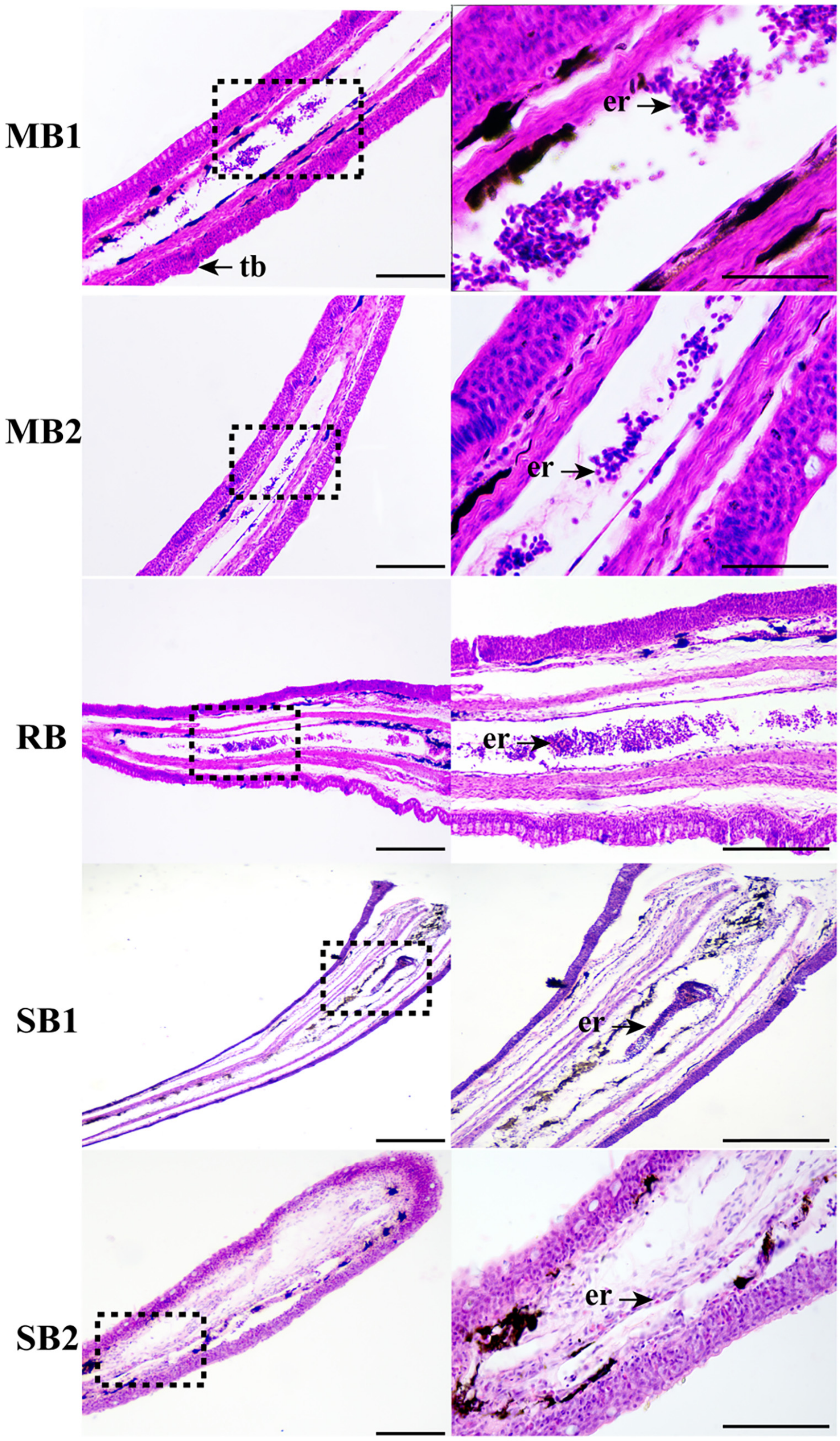


**Figure S1. Observations of H&E staining longitudinal sections of loach barbels.** MB, maxillary barbel; RB, rostal barbel; SB, submaxillary barbel; H&E, hematoxylin and eosin; tb, taste buds; er, erythrocytes. The visual fields in black dotted boxes were magnified nearby. The scale bars of the left and right column of longitudinal sections of loach barbels were respectively 30 μm and 100 μm.

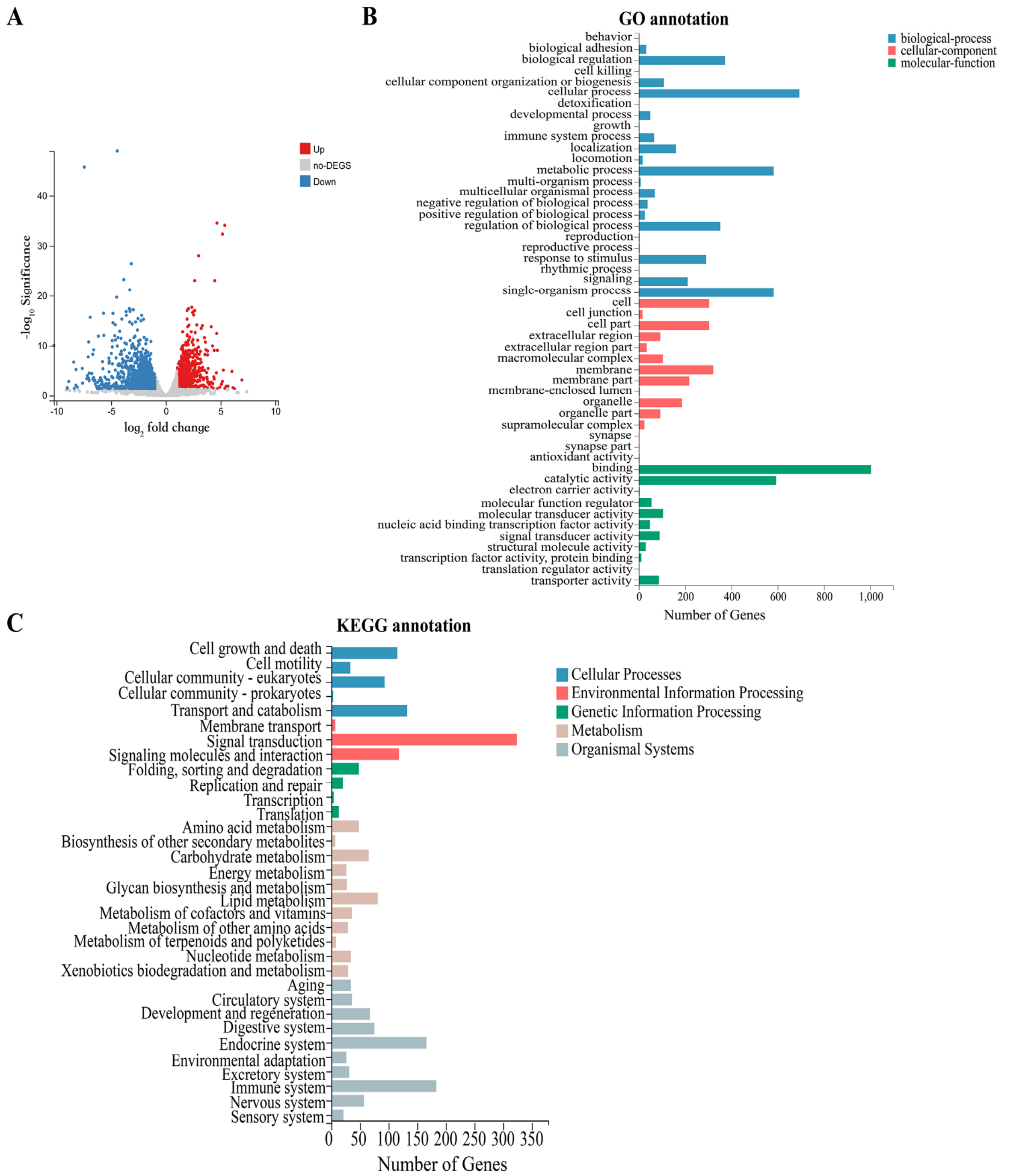


**Figure S2. Functional annotations of DEGs in barbels between T-XU and C-XU group (T-XU VS C-XU).** (A) Volcano map of DEGs between T-XU and C-XU group (T-XU VS C-XU). T-XU and C-XU group represented air exposure group and control group, respectively. (B) GO enrichment analysis of DEGs. (C) KEGG enrichment analysis of DEGs.

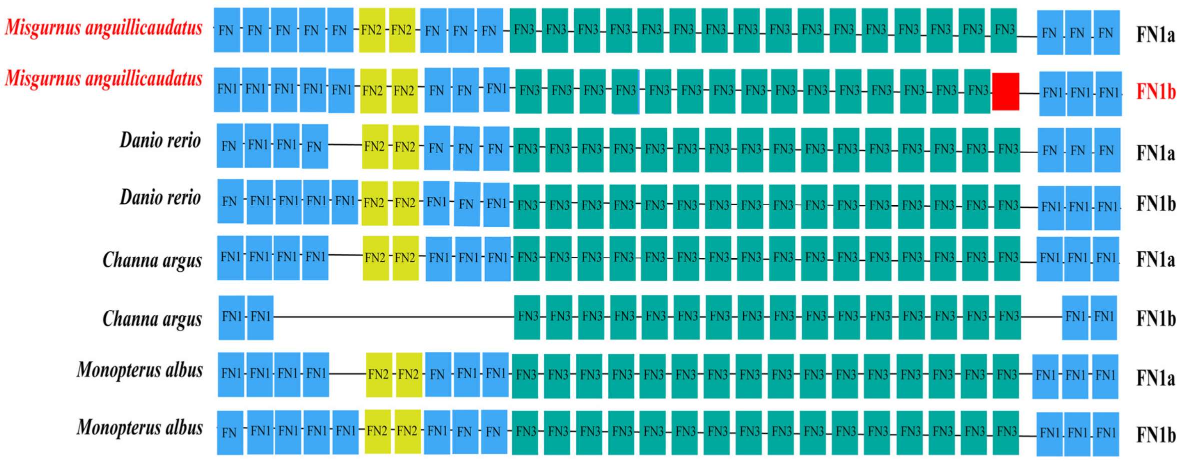


**Figure S3. Schematic diagram of the conserved domains of the deduced amino acid sequences of fibronectin 1 gene (*fn1*) in loach *M. anguillicaudatus*, compared with other three fish species (*D. rerio*, *C. argus* and *M. albus*).** The red box signified a lack of a type III repeat module (FN3) in the deduced amino acid sequences of *fn1b* gene of *M. anguillicaudatus*.

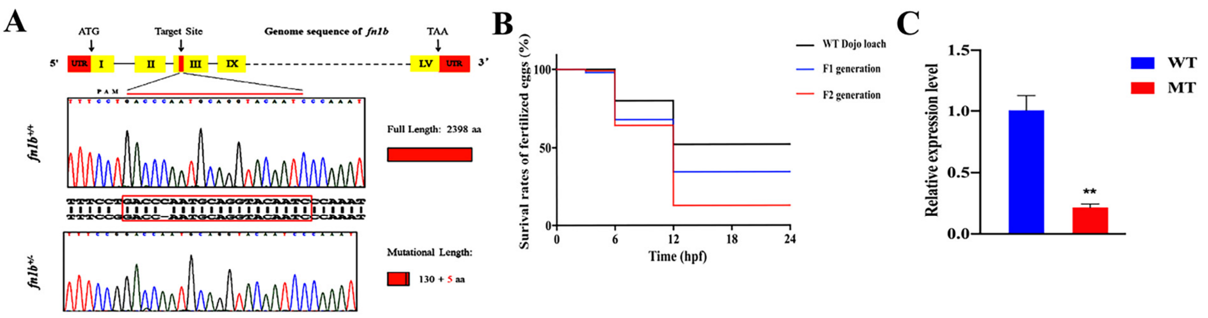

**Figure S4. *fn1b*-depletion loach produced by CRISPR/Cas9 technology.** (A) Schematic position of the CRISPR/Cas9 target site for *fn1b* gene knockout. A representative chromatogram of PCR-product sequencing of F1 generation in which indel mutations were presented. (B) A statistics of survival rates of fertilized eggs of wild-type loach (WT), F1 generation (F0 generation crossed with WT loach) and F2 generation (F1 generation self-crossed). (C) The transcription levels of *fn1b* in barbels of WT and *fn1b*-depletion loach (MT). ** indicated very significant difference ($p < 0.01$). hpf, hours post fertilization.

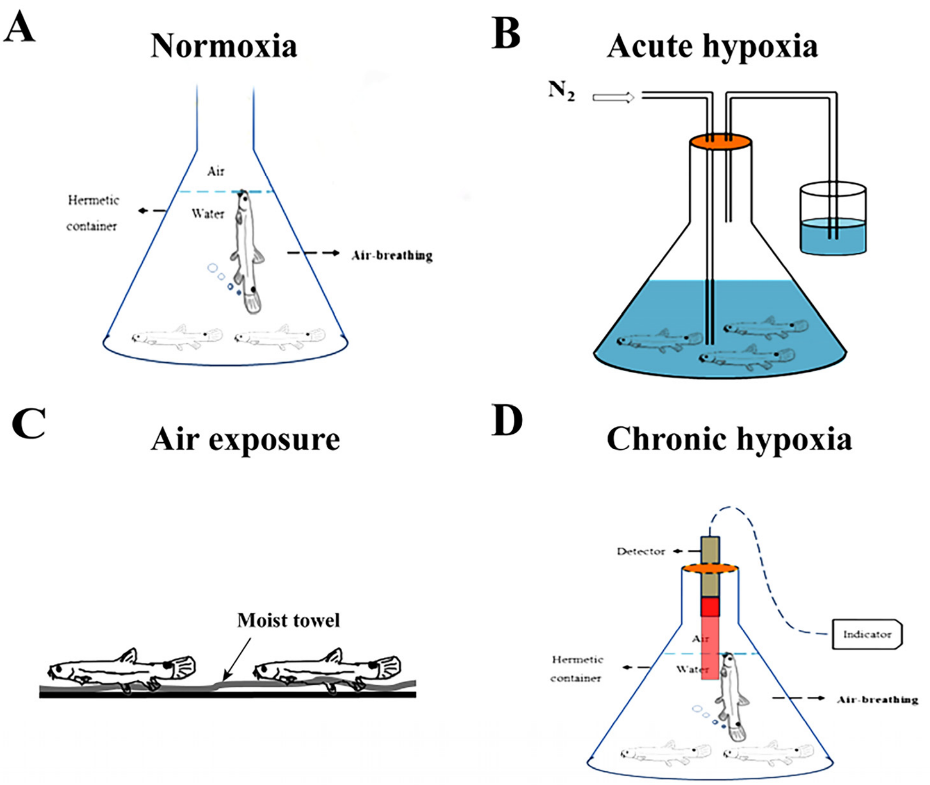


**Figure S5. Four treatments used in this study.** (A) Normoxia treatment device. (B) Acute hypoxia treatment device. One port of the hermetic conical flask was connected to flow $N_2$, and the other one to water, making the dissolved oxygen (DO) in the flask decreased sharply. (C) Air exposure treatment. The loach was placed on the moist towel for air exposure. (D) Chronic hypoxia treatment device. The loach was placed in a hermetic conical flask with DO level decreasing gradually and the water dissolved oxygen level was monitored by a detector.
